# Impact of Different Nutrition Strategies on Patients with Inflammatory Bowel Disease (IBD)

**DOI:** 10.3390/nu15041056

**Published:** 2023-02-20

**Authors:** Konstantinos Papadimitriou, Sousana K. Papadopoulou

**Affiliations:** 1 Faculty of Health and Rehabilitation Sciences, Metropolitan College, University of East London, Thessaloniki Campus, 54624 Thessaloniki, Greece; 2 Department of Nutritional Sciences and Dietetics, School of Health Sciences, International Hellenic University, 57001 Thessaloniki, Greece

In 1932, Burrill B. Crohn first published his report on a disease that mainly affects young adults by causing inflammation in the intestines and, specifically, the ileum [1]. Nowadays, inflammatory bowel disease (IBD) is recognized as a chronic inflammatory disease. This term refers to an entity of heterogeneous disorders including Crohn’s disease (CD) and ulcerative colitis (UC) [2].

To the best of our knowledge, the factors that activate IBD in young [3], middle-aged, and elderly people [4] are still unclear. It could be attributed to an interaction between genetic susceptibility and various unhealthy lifestyle factors such as smoking, alcohol consumption, low physical activity [5,6], high BMI (≥30 kg/m^2^) [7], and probably unbalanced nutritional habits [5]. Since nutrition relates to IBD onset and management, the aim of the present Special Issue, entitled “Impact of Different Nutrition Strategies on Patients with Inflammatory Bowel Disease (IBD)”, was to demonstrate the most recent nutrition prospects and strategies for inflammatory bowel disease patients.

IBD prevention through the research of different kinds of diets has the lion’s share in the international literature. In the present Special Issue, de Graaf et al. [8] investigated the pathophysiological factors and differences pertaining to diet in Dutch IBD and irritable bowel syndrome (IBS) patients. In a sample of 694 participants (238 with IBD, 261 with IBS, and 195 healthy controls), several dietary indices were estimated, i.e., the Dutch Healthy Diet Index 2015 (DHD 2015) and the Adapted Dietary Inflammatory Index (ADII). Additionally, fecal calprotectin was determined as a marker of intestinal inflammation and the Gastrointestinal Symptom Rating Scale (GSRS) was used as a measure of symptomatology. According to this study, both IBD and IBS patients demonstrated lower diet quality compared to controls. Specifically, they had lower values of DHD 2015 and lower consumption of dairy and high-fiber foods such as wholegrain products, fruit and vegetables, and legumes. However, based on ADII, there was no difference in the dietary inflammatory potential between IBD and IBS patients. Therefore, low diet quality was associated with higher intestinal inflammation, higher abdominal pain scores, and symptom scores in IBD and IBS patients, respectively. Thus, the authors highlighted the need for longitudinal studies to further elucidate the role of dietary factors in the development of symptom exacerbation [8].

A proteomic approach for the possible associations between dietary factors and circulating inflammatory proteins exacerbating IBD symptoms was presented by Bourgonje et al. [9]. Specifically, in this study, plasma concentrations of 73 different inflammation-related proteins were measured in 454 patients with IBD. In parallel, the dietary habits, body-mass index (BMI), smoking status, Montreal disease classification, Harvey–Bradshaw Index (HBI) for disease’s activity, the Simple Clinical Colitis Activity Index (SCCAI), and blood C-reactive protein (CRP) concentration were evaluated. The authors confirmed the association between different dietary patterns and immune-related plasma proteins in patients with IBD. Specifically, FGF-19, a gut-derived hormone, which regulates bile acid synthesis, was low in subjects following a diet rich in refined carbohydrates compared to subjects following the Mediterranean-style pattern. Moreover, a pattern high in alcohol and coffee intake was associated with increased levels of CCL11 and decreased IL12B, although the exact role of the latter in IBD is unclear [9]. Therefore, the exploration of many unexplored proteins in relation to specific foods or dietary patterns can possibly provide some answers about the IBD pathophysiology and management.

Amamou et al. [10] studied possible novel dietary therapies against intestinal fibrosis via aryl hydrocarbon receptor (AhR) ligands. The authors, through their in vitro study, tried to depict the effects of different dietary AhR ligands on intestinal fibrosis and, more specifically, the stimulation of TGF-1, which seems to trigger fibrotic mechanism in intestinal tissue. The authors revealed that none of the tested dietary AhR ligands such as FICZ, ITE, and kynurenine inhibited TGF-β-stimulated colonic fibroblasts. This lacking effect, according to the authors, may be due to incubation time, cell type, the fibroblastic process step, or the kind of AhR ligands chosen. Therefore, it is noted that further studies are required to examine the potential novelty of that method in intestinal fibrosis [10]. 

The macro- and micro-nutrient content of the diet is vital for the remission of IBD and constitutes a hot and multi-studied topic. In the present Special Issue, González-Torres et al. reviews the role of partial enteral nutrition [11] and Vernia et al. sheds light into the role of vitamin D intake [12] in IBD. To start with, González-Torres et al. [11] explored the effects of partial enteral nutrition, which is something fresh and new in the literature because it allows for the ingestion of some foods, compared to exclusive enteral nutrition. The literature shows that both methods have a high response, but the combination of partial enteral nutrition with a highly restrictive diet seems to have a high effectiveness in CD’s remission. Therefore, probably, partial enteral nutrition could be a better-tolerated alternative method, but further studies are necessary. 

Finally, Vernia et al. [12], in their review, searched for the effects of vitamin D intake on IBD. Generally, the authors quoted that vitamin D regulates the immune system, positively affecting intestinal homeostasis. Therefore, their review aimed to provide “the great picture” of the biological mechanisms of action and the therapeutic implications of vitamin D in IBD. According to in vitro and animal studies, it was found that vitamin D modulates the inflammatory response and affects the gut microbiome composition. In parallel, vitamin D deficiency is associated with disease activity and probably disease onset. In conclusion, the authors underline the potential role of vitamin D as a therapeutic agent, which they strongly encourage be investigated further.

This Special Issue provides novel knowledge on the complex nutrition strategies that contribute to IBD remission and decrease in symptoms severity, augmenting patients’ qualities of life. However, further studies are needed to shed light on this important topic and further finetune a personalized nutritional approach for patients with IBD.

## Data Availability

Not applicable.
